# *In vitro* screening of active flavonoid components from *B. javanica* flavonoids against protoscoleces of *Echinococcus granulosus* from sheep

**DOI:** 10.3389/fvets.2026.1763947

**Published:** 2026-06-24

**Authors:** Yueqi Yang, Jun Li, Jiayinaer Jikesanbayi, Kunlei Li, Bin Guo, Laizhen Liu, Meihe Hu, Songhan Liu, Jiahao Zhai, Xiaoying Wei, Luheng Xiao, Wenbao Zhang, Shaohua Zhai

**Affiliations:** 1 College of Veterinary Medicine, Xinjiang Agricultural University, Urumqi, Xinjiang, China; 2 Xin Jiang Key Laboratory of New Drug Researc and Development for Herbivorous Animals, Xinjiang Agricultural University, Urumqi, Xinjiang, China; 3 State Key Laboratory of Pathogenesis, Prevention and Treatment of High Incidence Diseases in Central Asia, Clinical Medicine Institute, The First Affiliated Hospital of Xinjiang Medical University, Urumqi, Xinjiang, China; 4 Yili Chuanning Biotechnology Co., Ltd., Yili, China

**Keywords:** *B. javanica* flavonoids, *Echinococcus granulosus*, metabolomics, molecular docking, proteomics

## Abstract

**Introduction:**

*Cystic echinococcosis*, caused by *Echinococcus granulosus* larvae, is a prevalent global zoonotic parasitic disease that primarily invades the liver. Current clinical treatments are limited by unsatisfactory therapeutic efficacy and obvious adverse side effects, creating an urgent demand for safe and effective alternative therapeutic agents. Brucea javanica flavonoids are natural active ingredients with potential antiparasitic properties, and this study aimed to explore their anti-protoscolex activity, biosafety, and underlying molecular mechanism against *E. granulosus*.

**Methods:**

The optimal extraction process of *B. javanica* flavonoids was optimized via ethanol extraction method. In *vitro* pharmacological experiments were performed to detect the inhibitory effect of flavonoid extracts on *E. granulosus* protoscoleces. Scanning electron microscopy and Caspase-3 activity assay were used to observe morphological damage and apoptotic activation of protoscoleces. CCK-8 assay and acute oral LD₅₀ test were conducted to evaluate biosafety, while HE staining was applied for pathological observation of major organs. An *E. granulosus*-infected mouse model was established to verify in vivo therapeutic efficacy, and HE as well as Masson staining were used to assess lesion repair. Furthermore, UHPLC-HRMS metabolomics, Astral DIA proteomics, and molecular docking were combined to screen core active flavonoid monomers, and in vitro and in vivo experiments were implemented to verify the activity of key monomers.

**Results:**

The optimal extraction conditions (80% ethanol, solid–liquid ratio of 1:20, 60 °C for 30 min) yielded a flavonoid content of 10.72%. The crude flavonoid extract exerted a significant concentration-dependent inhibitory effect on protoscoleces, with an inhibition rate of 85.88% at 40 mg/mL for 24 h. The extract severely damaged protoscolex ultrastructure and upregulated Caspase-3 activity by 4.22-fold to induce protoscolex apoptosis. Biosafety evaluation confirmed that 10 mg/mL flavonoids had no obvious host cell toxicity, with a mouse oral LD₅₀ of 1.43 g/kg and no pathological damage in major organs. In vivo results showed that 20 mg/kg flavonoid treatment effectively reduced hepatic cyst numbers and promoted fibrotic repair in mice. Multi-omics analysis identified 50 flavonoid components and 25 differentially expressed proteins enriched in antiviral immune and RNA degradation pathways, and 16 flavonoids with high target-binding affinity were screened. Among them, myricetrin was the only active monomer, and 5 mg/mL myricetrin glycoside treatment significantly reduced liver cyst volume and achieved effective parasite clearance and tissue repair in vivo.

**Discussion:**

This study systematically demonstrates the prominent anti-echinococcosis activity and good biosafety of B. javanica flavonoids both in vitro and in vivo. The flavonoids kill E. granulosus protoscoleces by destroying parasite structure and activating apoptotic pathways, and alleviate liver lesions by promoting tissue fibrosis repair. Myricetrin is identified as the core active substance responsible for the anti-parasitic effect. These findings reveal the material basis and potential mechanism of B. javanica against echinococcosis, filling the gap of natural anti-echinococcosis drug research. This work provides a reliable experimental basis for the development of novel safe and efficient anti-echinococcosis natural drugs, and offers a new strategy for the clinical treatment of cystic echinococcosis.

## 1 Introduction

Echinococcosis is a zoonotic parasitic disease caused by *Echinococcus granulosus* or *E. multilocularis*. The World Health Organization (WHO) classifies echinococcosis as one of the 17 neglected tropical diseases. Globally, about 1.0–5.7 million people are infected annually, with an economic burden exceeding US$ 3 billion per year (1–5). In Xinjiang, China, echinococcosis remains a severe public health concern with concern with high mortality are both high (6). Xinjiang is a major epidemic regin, with *E. granulosus* as the dominant pathogen, and cystic echinococcosis (CE) poses a persistent to regional zoonosis control and prevention (7).

The principal treatment options for CE are surgical and pharmaceutial interventions (8, 9). Benzimidazoles (e.g., albendazole, mebendazole) are first-line drugs recommended by WHO guidelines. Benzimidazoles show low aqueous solubility (< 0.5 μg/mL), poor oral absorption (bioavailability < 5%), and obvious hepatotoxicity and gastrointestinal side effects after long-term high-dose use (10, 11). In recent years, various synthetic and herbal compounds have been explored against CE, including flubendazole, metformin, tacrolimus (12–14); and plant extracts from Rhamnus spp., *Hippophae rhamnoides*, and Lindera spp (15–17). Despite these advances, safe, highly effective, and resistance-resistant therapeutic agents are still urgently needed for clinical CE management.

*B. javanica* flavonoids is a traditional medicinal plant, and its dried ripe fruit is used clinically. Modern pharmacological studies indicate that *B. javanica* has significant anti-tumor (18–20) and anti-inflammatory activities (21–23). For antiparasitic effects, *B. javanica* extracts show potent anti-malarial activity against *Plasmodium falciparum*, and oral or intramuscular administration reduces parasitemia (24, 25), its ethanol extract also exerts anticoccidial effects at specific concentrations (26). Flavonoids are polyphenolic compounds with broad-spectrum bioactivities. Flavonoids exert antiparasitic effects by binding to functional proteins, regulating apoptotic signaling, and disrupting energy metabolism (27).

This study optimized flavonoid extraction, evaluated *in vitro* and *in vivo* efficacy and safety, and used metabolomics, proteomics, and molecular docking to screen active components and clarify mechanisms. We hypothesized that *B. javanica* flavonoids kill *E. granulosus* protoscoleces by activating apoptosis and regulating RNA metabolism pathways, with myricetrin as the key active monomer.

## 2 Materials and methods

### 2.1 Ethical norms statement

The Animal Experimental Ethics Committee of Xinjiang Agricultural University approved all animal experiments. All mice were housed in clean cages within a temperature- and humidity-controlled room and provided with standard laboratory chow and purified water ad libitum.

Anesthesia was induced using pentobarbital sodium (50 mg/kg). To ensure animal welfare and alleviate suffering, euthanasia was carried out by cervical dislocation following the induction of deep anaesthesia.

### 2.2 Extraction process of flavonoids from *B. javanica*

*B. javanica* (batch No. BJ-20220815, 50.00 g) was ground and crushed. Petroleum ether (boiling point 60–90 °C) was added at the solid–liquid ratio of 1 6 (w/v). The system was magnetically stirred at 25 °C for 10 min. After standing for 24 h and vacuum-filtering, the residues were collected and air-dried to obtain the defatted material. The ethanol solution at a set concentration was added and placed in a numerical control ultrasonic extractor for dynamic extraction at frequency 80 kHz and power 1 kW. Then after vacuum-filtering, the filtrate was concentrated to about 50 mL and then freeze-dried to obtain the crude flavonoid extract.

### 2.3 Determination of purity of total flavonoids extract

Rutin was used to establish the standard curve for flavonoid determination. Crude extract (10 mg) of flavonoids from *B. javanica* was dissolved in 100 mL of 60% ethanol. Then 1 mL of the solution was removed to a 25 mL volumetric flask, to which 60% ethanol solution was added. The resulting solution was stirred, dissolved and colored according to the color development step of the NaNO_2_-AlCl_3_-NaOH method. After that, the solution was transferred to a color development plate, and the absorbance of each repeated group solution was determined at 510 nm. Purity (%) was calculated as (absorbance of diluted sample × volume of sample solution × dilution multiple)/total mass × 100.

### 2.4 Isolation and *in vitro* culture of protoscoleces

Metacercariae-infected sheep livers were collected from the Hualing Slaughterhouse in Urumqi, Xinjiang. The cyst fluid containing the protocercariae was extracted from the liver cysts and transferred to sterile centrifuge tubes. When the protoscoleces were static, the supernatant was removed, and the protoscoleces of the white particles in the precipitates were retained. Then the precipitates were washed several times with PBS with 1% double antibody. After the tissue fragments were removed, RPMI-1640 with 10% FBS was added, and the cells were grown in a 5% CO₂ incubator at 37 °C for 24 h. The activity of protoscoleces was detected by eosin staining, and the protoscoleces with activity greater than 95% were subjected to subsequent experiments.

### 2.5 Activity test of *B. javanica* flavonoids on protoscoleces *in vitro*

Protoscoleces (100 μL, 1,000 /mL) were seeded into 96-well plates. Crude *B. javanica* flavonoid extract was added at final concentrations of 10, 20, 40 mg/mL The plates were cultured for up to 72 h at 37 °C in a 5% CO_2_ atmosphere. The activity of protoscoleces after drug action was detected by eosin staining, and the mortality rate was calculated on SPSS 17.0.1.

### 2.6 Detection of Caspase-3 level in protoscoleces apoptosis

After the protoscoleces were treated with drugs (10, 20, or 40 mg/mL), they were treated with lysis buffer, gently stirred and incubated on ice for 30 min. The cell lysate was centrifuged at a high speed (12,000 rpm, 10 min) at 4 °C, and the supernatant was collected to avoid precipitation. According to the kit instructions, the cell lysate was mixed with the Caspase-3 substrate and added to a 96-well plate and incubated in a buffer (37 °C, 30–60 min) with 6 replicates in each group. Determination of Caspase-3 The absorbance was measured at 405 nm using a microplate reader to evaluate the effect of drug treatment on Caspase-3 activity.

### 2.7 Observation of scanning electron microscope morphology of protoscoleces treated with *B. javanica* flavonoids

The cleaned protoscoleces (1,000/mL) were added with 40, 20, and 10 mg/mL crude extract of *B. javanica* flavonoids, and cultured for 0, 6, 12, 24, or 48 h. During the culturing, the morphological changes of protoscoleces were observed regularly under inverted microscopy, and their vitality and growth were recorded. The protoscoleces after drug action were fixed with FAA fixative for 24 h. The fixed protoscoleces were dehydrated in 30, 50, 70, 90, 95, or 100% ethanol for 20 min. After dehydration, liquid tert-butanol was added to each sample tube. The dehydrated protoscoleces were dried in a freeze dryer for 8 h. The dried protoscoleces were fixed on the conductive adhesive on the sample table for ion sputtering. The treatment conditions were: current 20 mA, time 240 s. The morphological changes of protoscoleces were tested with Quanta 250 FEG field-emission SEM.

### 2.8 *Cytotoxicity of B. javanica* flavonoids

The passaged BHK-21 cells were digested with trypsin, diluted to 1 × 10^6^ cells/mL, and inoculated in 96-well plates (100 μL/well). The crude extract of flavonoids from *B. javanica* was added to 96-well plates at 10, 20, or 40 mg/mL (100 μL/well). At the same time, a negative control group was set up and incubated at 37 °C in 5% CO_2_ incubator for 0, 6, 12, 24, or 48 h. After that, 10 μL of a CCK-8 reagent was added to each well, mixed by gentling shakening the 96-well plates, and then cultured at 37 °C for 2 h. Next, the absorbance (OD) of each well was detected at 450 nm using the microplate reader. The cell survival rate (%) was computed as (OD treatment group-OD blank group)/(OD control group-OD blank group) × 100%. The cell survival curve was drawn with the drug concentration as the abscissa and the cell survival rate as the ordinate, and the toxicity curve was calculated using GraphPad Prism.

### 2.9 LD_50_ of *B. javanica* flavonoids crude extract in mice

Forty healthy mice (20 ± 5 g) were separated randomly and evenly into 4 groups. *B. javanica* flavonoids were intraperitoneally injected to the mice at 2.45, 1.875, 1.35, and 1 g/kg. The injection dose was 0.2 mL/10 g body weight. The survival status and death of the mice were recorded every 2 h within 72 h of administration. The heart, liver, spleen, kidney and brain of each dead mouse were fixed with 10% neutral formaldehyde, and sections were prepared by HE staining to observe the pathological changes of each organ. LD_50_ was calculated as: logLD_50_ = X − i (Zp − 0.5), and the standard error SlogLD_50_ = i × √[(∑p − ∑p2)/(n − 1)], where X is the logarithm of the maximum dose, i is the logarithmic interval of the dose, Zp is the probability unit corresponding to the mortality of each dose group, p is the mortality rate, and n is the number of experimental animals.

### 2.10 Masson cytochemistry staining

The paraffin sections of liver tissues from the model mice were dewaxed and rehydrated, and the morphology of the liver cyst fiber tissue was observed according to a Masson staining kit (Suolaibao, G1340, China).

### 2.11 Model test of *B. javanica* flavonoids crude extract in treatment protoscoleces infection in mice

Mice were anesthetized, and livers were exposed under laparotomy. The protoscoleces (5,000/mL) were taken and injected into the liver lobe tissue at 45 °. Immediately after the injection, a cotton swab was pressed to stop bleeding, and the peritoneum, abdominal wall, and skin were sutured. After 7 days of model establishment, 40, 20, or 10 mg/kg *B. javanica* flavonoids were injected intraperitoneally into the mice for 21 days (*n* = 6). At the end, the mice were sacrificed by cervical dislocation, and the livers were removed after dissection. The livers were fixed with 10% neutral formaldehyde, and sections were made by HE staining and Masson staining to observe the morphological and structural changes of liver cysts.

### 2.12 Metabolomics analysis of *B. javanica* flavonoids on protoscoleces *in vitro*

The protoscoleces (1,000/mL), which were isolated *in vitro,* were added to 96-well plates. Then, *B. javanica* flavonoids (10 mg/mL) were added to each well. The normal cultured protoscoleces were adopted as a negative control, and the crude extract of *B. javanica* flavonoids served as a drug component control. When the mortality rate of protoscoleces reached 30%, the protoscoleces were collected, washed repeatedly with sterile PBS to remove the residual liquids, and centrifuged using a freeze centrifuge. After discarding excessive liquids, the protoscoleces were collected and weighed to ensure that each sample was no less than 200 mg. Totally 3 samples were collected, and all samples were placed in a cryopreservation tube. UHPLC-HRMS was conducted.

The samples were separated using an ultra-high performance liquid chromatography (UHPLC) system (Vanquish, Thermo Fisher Scientific, Bremen, Germany), combined with an ACQUITY UPLC HSS T3 column (2.1 mm × 100 mm, 1.8 μm). The column temperature was set to 35 °C and the flow rate to 0.3 mL/min. The mobile phase consisted of A: 0.1% formic acid in water, and B: 0.1% formic acid in acetonitrile. The Q-Exactive HFX mass spectrometer was then used to collect the primary and secondary mass spectra of the samples. It was coupled with the UHPLC system and mass spectrometry was performed in both positive and negative electrospray ionization (ESI) modes. The spray voltage was set to 3,800 V (ESI+)/3,500 V (ESI-), the sheath gas pressure to 45 arb, the auxiliary gas pressure to 20 arb, the ion transfer tube temperature to 320 °C and the nebulizer temperature to 350 °C. The detection mode was full scan/data-dependent secondary scan (full MS/dd MS2), with a resolution of 60,000 for the primary scan and 15,000 for the secondary scan. The top 10 MS1 ions were selected for MS/MS spectra and collision energies (CEs) were set at stepwise normalized energy levels of 20, 40, and 60. The primary mass-to-charge ratio scanning range was 90–1,300. Six microlitres of blank group samples, *B. javanica* solution and blank group + *B. javanica* flavonoids samples were aspirated precisely for LC–MS analysis. Each batch of blank and drug administration group samples was injected once, while blank + *B. javanica* flavonoids samples were injected three times and *B. javanica* flavonoids samples five times.

### 2.13 Proteomics analysis of *B. javanica* flavonoids acting on protoscoleces *in vitro*

Protoscoleces were homogenized, precipitated with TCA/acetone, and then lysed with SDT buffer. After sonication and boiling lysis, the supernatant was collected by centrifugation and quantified using the BCA assay. A 15 μg of protein was mixed with 5 × loading buffer, boiled, separated by 4–20% SDS-PAGE, and stained with Coomassie brilliant blue. Equal amounts of protein from each sample were pooled to prepare a QC sample. Protein digestion was performed using the FASP method: reduction with DTT, alkylation with IAA, buffer exchange via ultrafiltration, and overnight digestion with trypsin. Peptides were desalted using a C18 cartridge, concentrated by vacuum centrifugation, reconstituted in 0.1% formic acid, spiked with iRT calibration peptides, and subjected to DIA mass spectrometry analysis.

### 2.14 Molecular docking

The binding mode and affinity of small-molecule ligands with proteins were predicted via molecular docking. The molecular docking software AutoDock and related tools (MGLTools and PyMOL) were installed, and the three-dimensional structure data of proteins and small molecules were obtained from Uniprot and PubChem. Protein structures were prepared by removing water molecules and co-factors, adding hydrogen atoms and Gasteiger charges, and saving in PDBQT format. Small molecules were energy-minimized, and added with hydrogen atoms and charges. Rotatable bonds were defined, and ligands were also saved in PDBQT format. The docking grid box was defined to encompass the known active site of the protein or based on a homologous structure, with appropriate box dimensions and grid point spacing set. The molecular docking calculation was started after AutoDockVina generated the input file, and the results were outputted in the form of binding energy and visual conformation. Finally, the binding energy was extracted, and together with the molecular docking results, was visualized on PyMOL.

### 2.15 Statistical analysis

Statistical analysis and plotting were performed using GraphPad Prism (version 9.5.0); Differences were assessed by two-tailed Student’s *t*-test or one-way ANOVA followed by Tukey’s *post-hoc* test. Normality was confirmed by Shapiro–Wilk test before ANOVA, and p -values were displayed in the GP format (***p < 0.001, **p < 0.01, *p < 0.05, *ns* ≥ 0.05). All graphical data wereare expressed as mean ± SD.

## 3 Results

### 3.1 Measurement of minimum effective concentration of *B. javanica* flavonoids on protoscoleces *in vitro*

**Figure 1:**
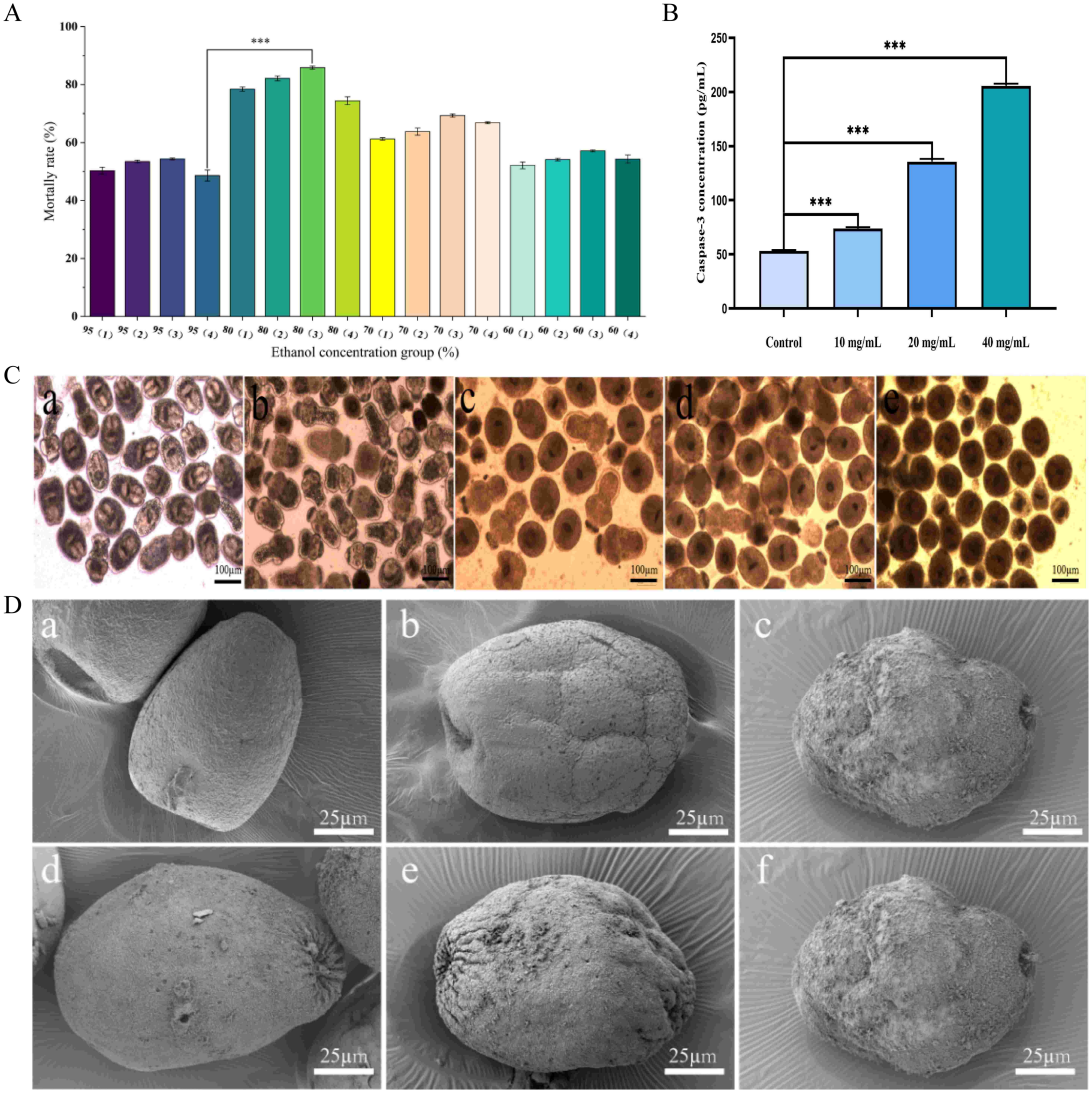
**(A)** Relationship between ethanol concentration and inhibition rate of primary cysts. **(B)** Apoptosis of primary cysticercus detected using Caspase-3 ELISA kit. **(C)** Optimal drug conditions under light microscopy for 24 h, protoscolece morphology 100x [a–e: 0 (blank), 5, 10, 20, 40 mg/mL respectively]. **(D)** Optimal drug conditions for 24 h protoscoleces morphology 2,000x under SEM (a blank group, b 6 h, c 12 h, d 24 h, e 48 h, f 72 h).

The inhibitory effect of *B. javanica* flavonoids at different concentrations on protoscoleces was evaluated *in vitro*. With an optimized extraction process (80% ethanol, solid–liquid ratio = 1:20, 60 °C for 30 min) and with rutin as the standard, the purity of the *B. javanica* flavonoids was determined to be 10.72% (Figure 1A). The extract obtained under these optimized conditions exhibited a strong inhibitory effect on protoscoleces, with an inhibition rate of 85.88 ± 0.053% (Figure 1B). Different concentrations of flavonoid extracts from *B. javanica* (40, 20, 10, 5 mg/mL) were used to act on PSCs *in vitro* for 24 h. Compared with the untreated control group (Figure 1C(a)), at 5 mg/mL, the body surface was slightly rough and the activity was slow (Figure 1C(b)). At 10 mg/mL, the body surface was rough, slightly contracted and slow in activity (Figure 1C(c)). At 20 mg/mL, shrinkage collapse, structural blur and cell rupture occurred (Figure 1C(d)). The level at 40 mg/mL caused the body wall to disintegrate, and the surface completely lost smoothness, showing serious collapse and concave-convex morphology (Figure 1C(e)). The morphological damage to protoscoleces was also time-dependent under treatment with the 40 mg/mL extract. Compared to the intact and smooth untreated control (Figure 1D(a)), surface cracks appeared at 6 h (Figure 1D(b)). These cracks increased and were accompanied by depressions at 12 h (Figure 1D(c)). The damage was intensified at 24 h, with significantly increased surface roughness, cracking and depression (Figure 1D(d)). By 48 h, the worm body was damaged, and the sucker slightly deformed (Figure 1D(e)). Severe damage to the worm body and complete sucker deformation wereobserved at 72 h (Figure 1D(f)). After 12 h, the activity of Caspase-3 in the protoscoleces of *E. granulosus* increased concentration-dependently significantly with the rise of treatment concentration. The activity of Caspase-3 in the high-content group was much higher than in the control group and the low-content group (*p* < 0.05), suggesting that high-content treatment could significantly promote the apoptosis of protoscoleces.

### 3.2 *B. javanica* flavonoids *in vivo* drug effect and drug safety evaluation observation

**Figure 2:**
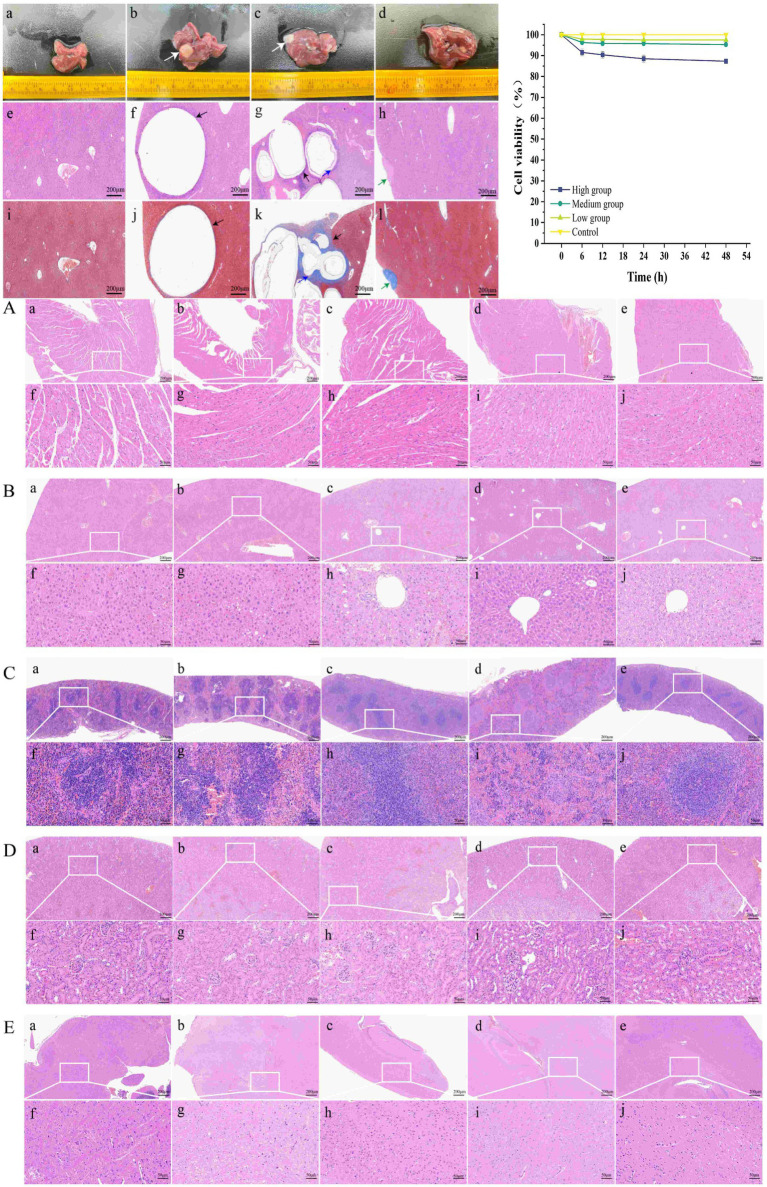
**(A)** Crude extract of *B. javanica* flavonoids in treatment of protoscoleces-infected mouse model autopsy (a–d) pathological sections HE staining sections (e–h) 40 × and Masson staining sections (i–l) 40×. **(B)** *B. javanica* flavonoids crude extract CCK-8 cytotoxicity experiment. **(C)** Mice LD50 visceral organs HE staining sections, **(A–E)** for different experimental groups, (a–e) 40×, (f–j) 200 × .

The crude extract of *B. javanica* flavonoids at 10 mg/kg significantly reduced the cyst volume of the model mice, accompanied by a mild fibrotic reaction locally, indicating this concentration could effectively eliminate primary cercariae and promote local repair. The 20 mg/kg drug concentration showed the most obvious effect, almost completely eliminating the cysts, and a significant fibrotic reaction occurred in the local tissues, suggesting a strong tissue repair effect (Figure 2A). The CCK-8 test after treating the cells with the crude extract of *B. javanica* flavonoids for 48 h showed that the cell survival rates in the medium- and low-concentration groups (10 and 5 mg/mL) remained above 95%, indicating the toxicity of the flavone extract on the cells was relatively small and did not significantly inhibit cell growth and survival (Figure 2B). The half-lethal dose of the drug in the mice was calculated to be 1.43 g/kg. Pathological section observations of organs and tissues in the mice (heart, liver, spleen, lung, kidney, and brain) showed that all tissues presented normal cell structures and tissue architecture, with no sign of edema, necrosis, inflammatory infiltration, or other pathological damages (Figure 2C).

### 3.3 Number of identified compounds in TCM and its administration group samples

The compounds were identified by searching the local HRMS database of TCM. The first-order mass error was below 25 ppm, and the match grade of the second-order fragmentation spectrum exceeded 0.7. The chemical components in the specimens of TCM and its administration group were tested and found out. Statistical results are listed in Table 1. Among them, 2,143 compounds were identified in the positive ion mode (POS), and 1,266 *B. javanica* flavonoids entered the protoscoleces. Totally 1,607 species were found out in the negative ion mode (NEG), and 958 species entered the protoscoleces. In the POS and NEG together, 3,482 substances were identified, and 2,093 *B. javanica* flavonoids were in the protoscoleces.

**Figure 3:**
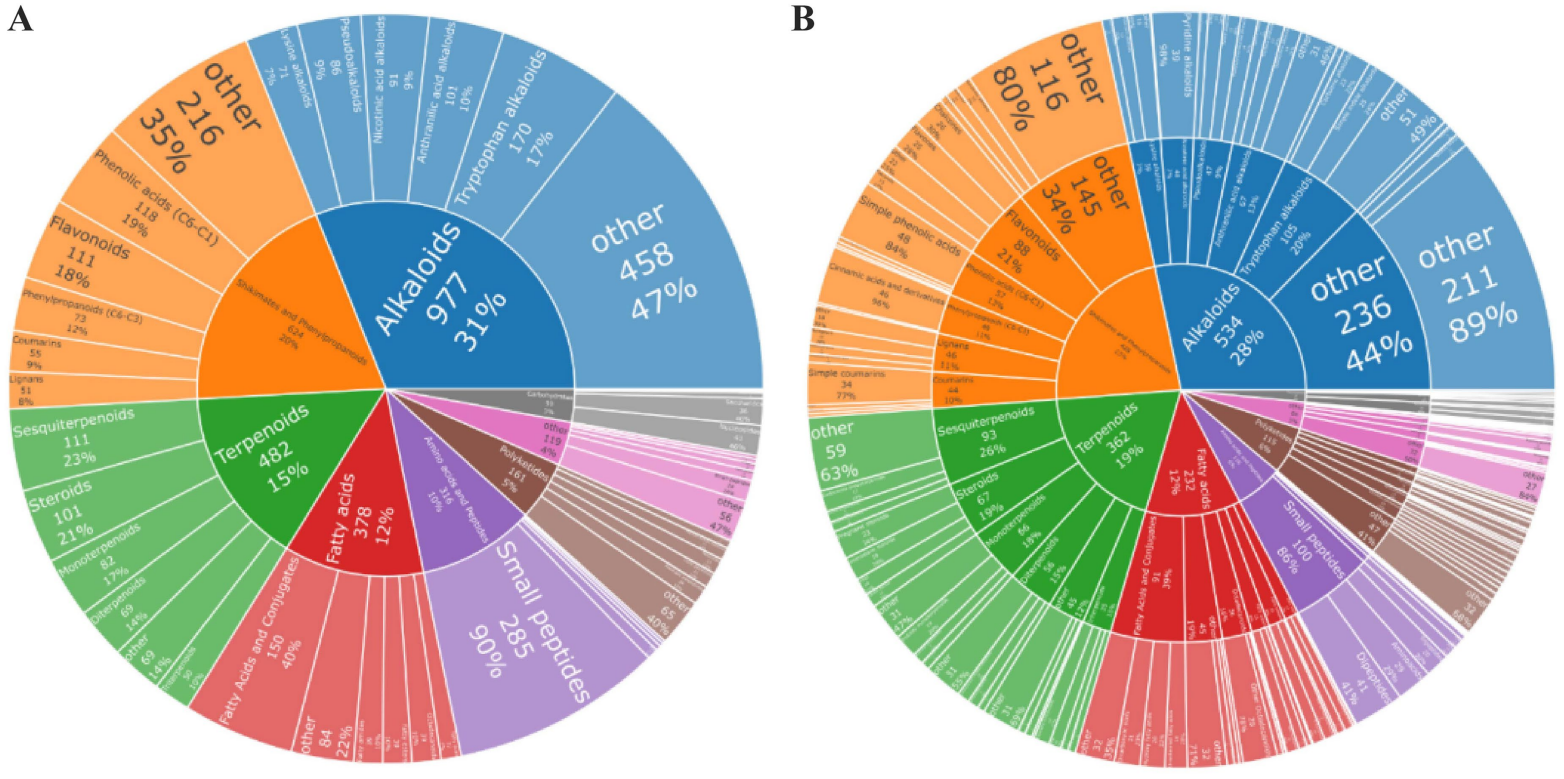
**(A)** Proportion of flavonoids in *B. javanica* flavonoids in each chemical classification. **(B)** Proportion of flavonoids in *B. javanica* flavonoids in amount of protoscoleces accounting for.

**Figure 4:**
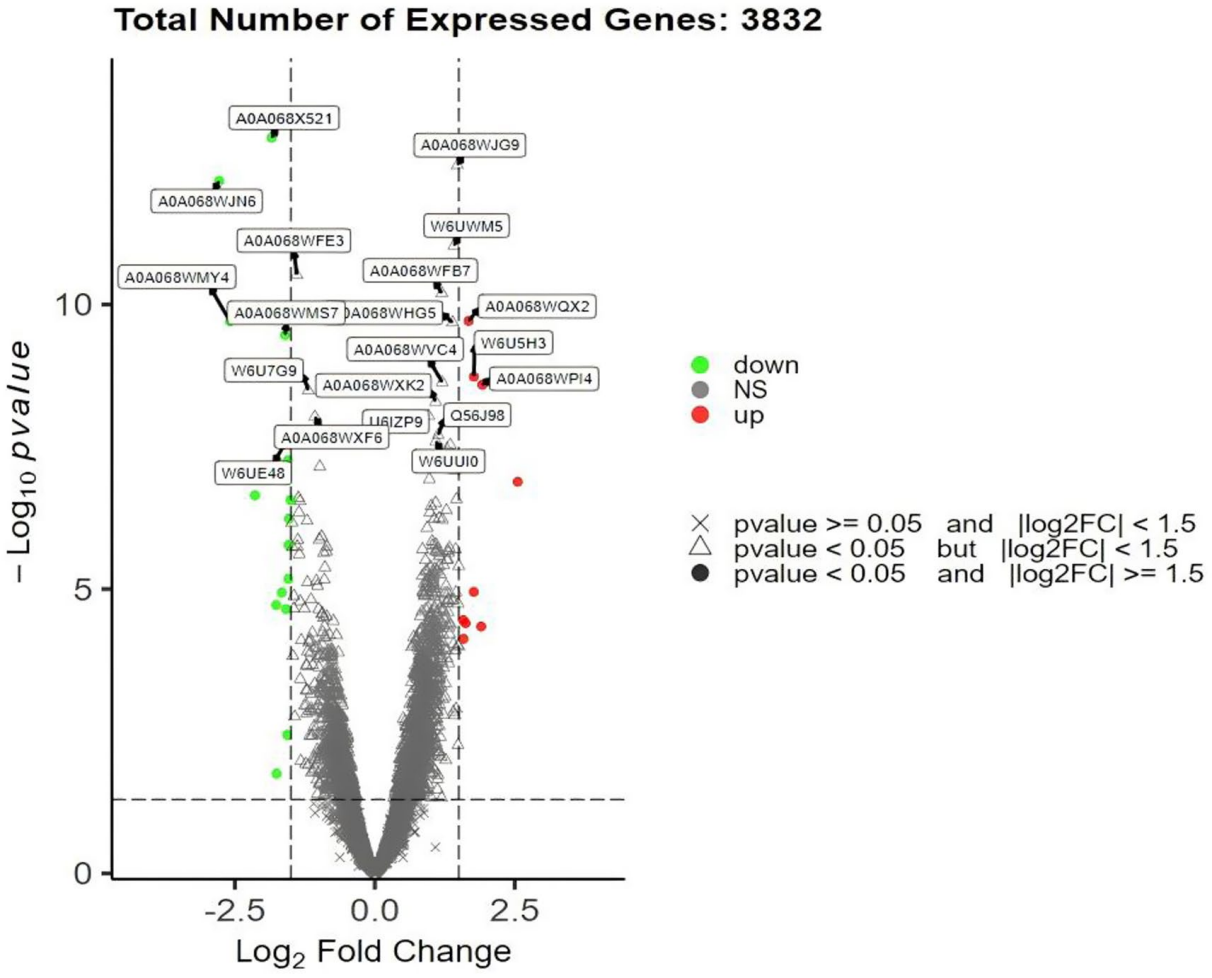
Differential protein volcano map.

**Figure 5:**
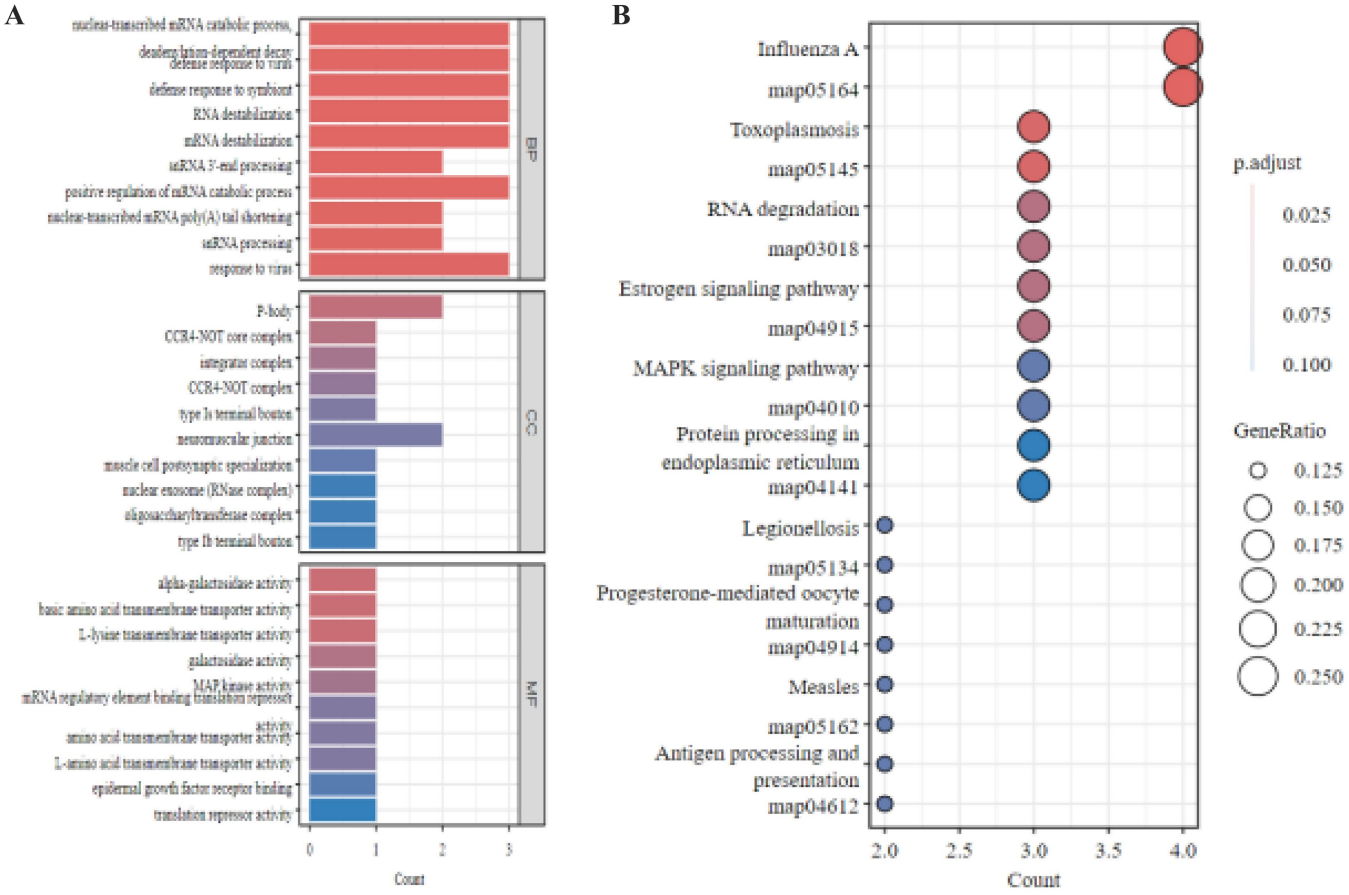
**(A)** Bar chart of GO enrichment and **(B)** bubble chart of KEGG enrichment for candidate proteins.

**Figure 6:**
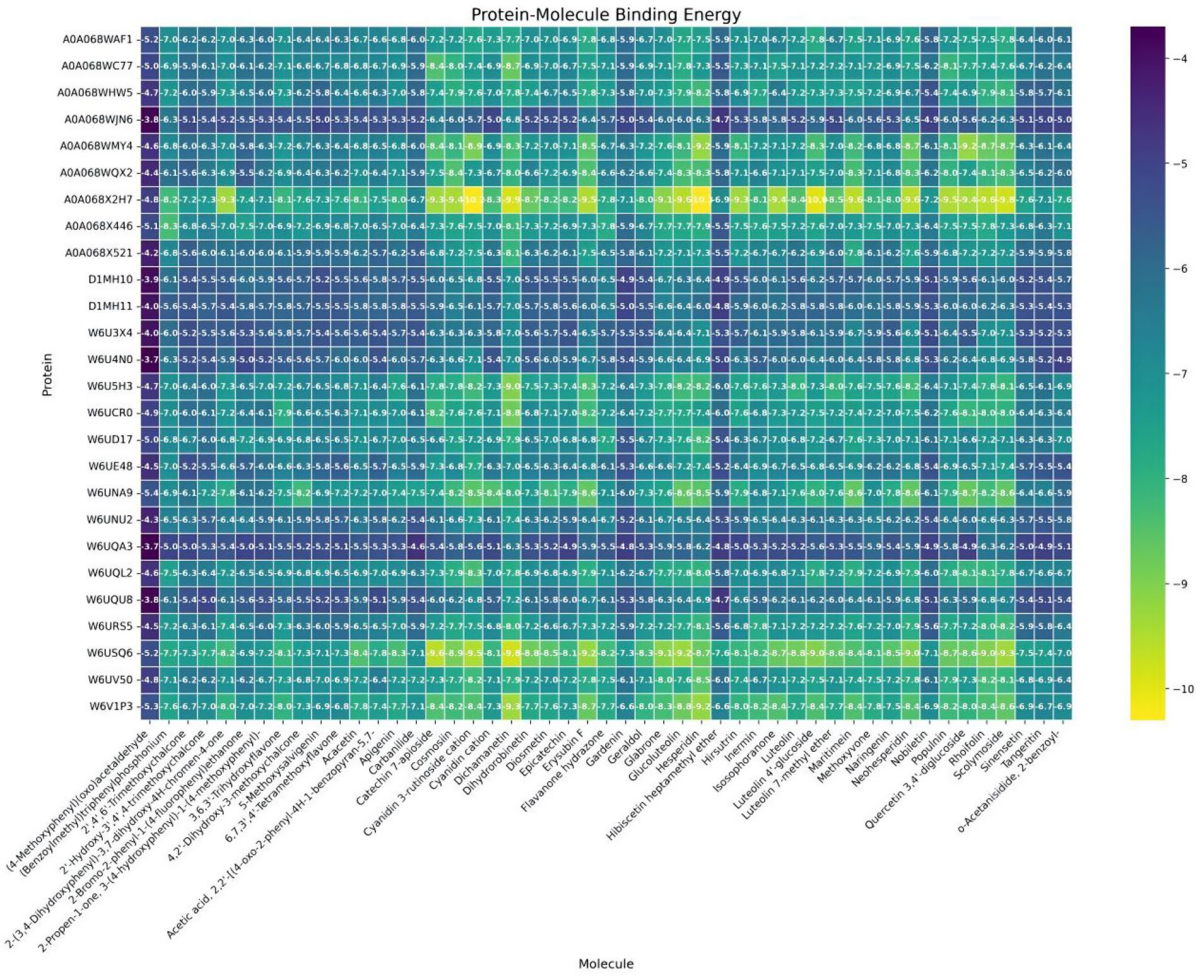
Molecular docking results of flavonoids and differential proteins.

Classification of the identified compounds revealed the following composition: alkaloids (31%), shikimates and phenylpropanoids (20%), terpenoids (15%), fatty acids (12%), amino acids and derivatives (11%), and small peptides (9%). The proportions of compounds classified into tissues are shown in Figures 4–6. Alkaloids, Shikimates and phenylpropanoids, Terpenoids, Fatty acids, Small peptides, and Amino acids and derivatives account for 31, 21, 15, 12, 6, and 6% respectively (Figure 3; Table 1).

**Table 1:** Identified number of compounds in positive and negative ion modes.

Detection ion mode	Number of flavonoids identified in *B. javanica* flavonoids	Number of flavonoids from *B. javanica* flavonoids affecting protoscoleces
POS	2,143	1,266
NEG	1,607	958
Total	3,482	2,093

### 3.4 Number of compounds identified of TCM and its administration group samples

Totally 50 flavonoids were recognized by POS mass spectrometry. The matching scores (Score) of all compounds were higher than 0.9000, indicating the identification results have high confidence. These compounds mainly include flavonoid glycosides, aglycones and methoxylated flavonoids as well as small amounts of chalcone derivatives and other modified flavonoid structures. Among them, the matching degrees of cosmoside, luteoloside and luteolin were close to a full score, suggesting their structural characteristics are highly consistent with the database. In addition, atypical derivatives were detected, which may be related to sample pretreatment or pyrolysis by-products. The data revealed the diversity of flavonoids in the samples, and especially the high-score components may be the key active substances. Nevertheless, it is necessary to combine NEG and secondary mass spectrometry to further exclude duplicate items and confirm the structural uniqueness (Table 2).

**Table 2:** Flavonoids identified in positive ion mode.

Molecular name	No.	m/z	RT min	Formula	ppm	adduct	score	PubCHEM	CAS
Cosmosiin	M433T336	433.1133	5.6	C_21_H_20_O_10_	0.8	[M + H]+	0.9999	5,280,704	578–74-5
Luteolin	M285T336	285.0409	5.61	C₁₅H₁₁O₆	2.5	[M-H]+	0.9998	5,280,445	491–70-3
Apigenin	M269T495	269.0461	8.25	C₁₅H₁₀O₅	1.5	[M-H]+	0.9996	5,280,443	520–36-5
Gardenin	M419T204_2	419.1317	3.4	C_21_H_22_O_9_	4.8	[M-H]+	0.9985	261,859	21,187–73-5
myricetrin	M403T642	465.103	10.7	C_21_H_20_O_12_	1.1	[M-H]+	0.9977	5,281,673	17,912–87-7
Glucoluteolin	M447T295	447.094	4.92	C₂₁H₂₀O₁₁	0.7	[M-H]-	0.9972	5,280,637	5,373–11–5
Acacetin	M283T6691	283.0681	11.15	C₁₆H₁₂O₅	2.2	[M-H]^−^	0.9971	5,280,442	480–44-4
Geraldol	M299T605	299.0565	10.08	C₁₆H₁₂O₆	1.5	[M-H]^−^	0.9971	5,482,101	21,511–25-1
Acacetin	M283T669_1	283.0681	11.15	C_16_H_12_O_5_	2.2	[M-H]^−^	0.9971	5,280,442	480–44-4
Luteolin 4′-glucoside	M447T342_1	447.094	5.7	C_21_H_20_O_11_	0.7	[M-H]^−^	0.9957	5,319,116	6,920-38-3

### 3.5 Differentially expressed protein analysis

**Figure 7:**
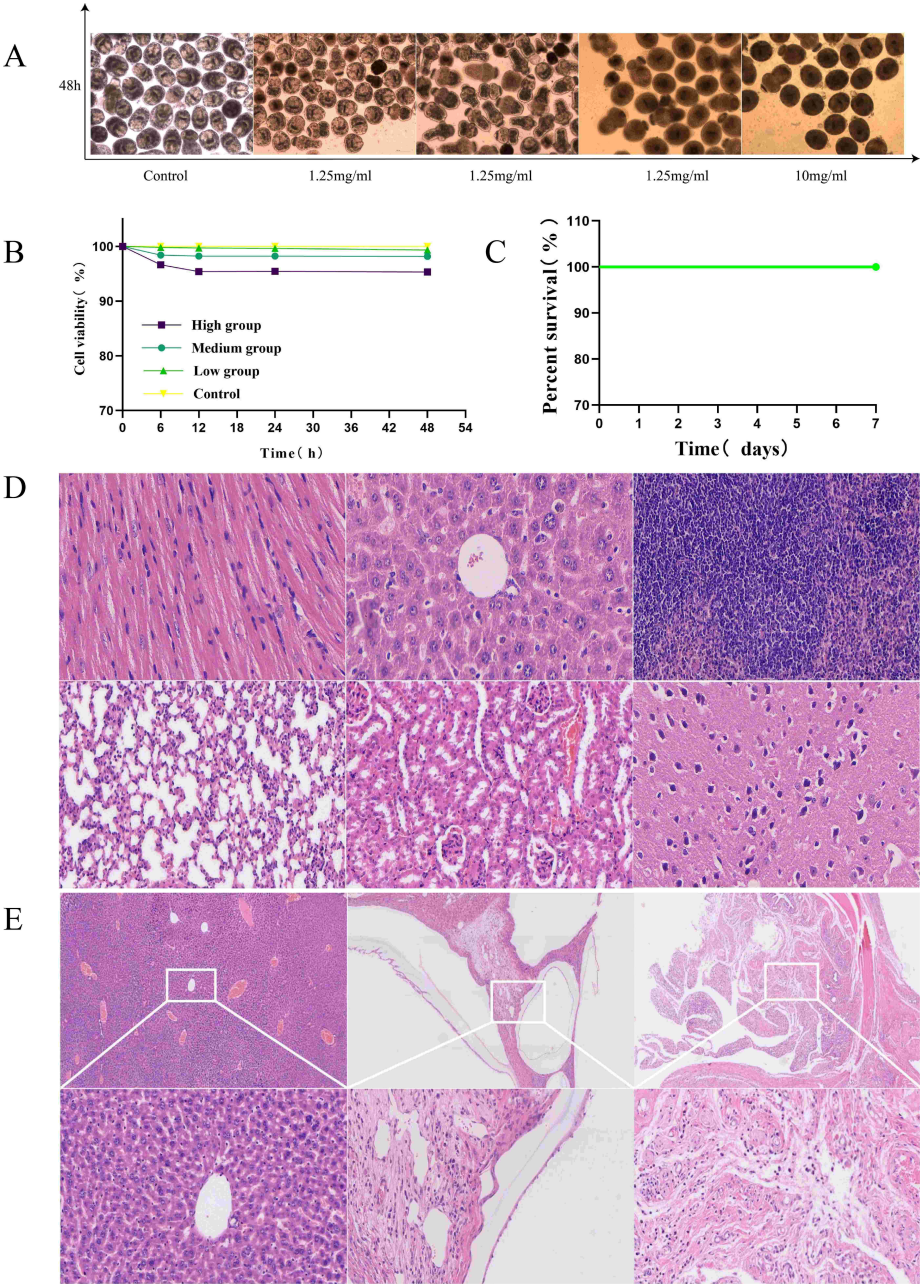
**(A)** Effects of different myricetrin concentrations on protoscoleces. **(B)** CCK-8 cytotoxicity assay of myricetrin. **(C)** Survival rate, and **(D)** HE staining sections of various tissues and organs in mice injected with 100 mg/mL myricetrin. **(E)** HE staining sections of various tissues and organs of mice in control group, model group, and treatment group.

Totally 3,822 proteins were identified from DESeq2 analysis. There were 25 DEPs (9 up-regulated and 16 down-regulated) between the blank group and the brucea flavonoid group. GO enrichment showed that *B. javanica* flavonoids acted through multi-dimensional mechanisms. In the biological process (BP), they significantly activated the “response to virus” mediated by the interferon signaling pathway and dynamically regulated “mRNA destabilization.” At the cellular component (CC) level, the DEPs were enriched in the RNA metabolic core units “integrator complex” and “P-body,” suggesting the degradation complex targeted to destroy the stability of parasite mRNA. On the molecular function (MF), the enrichment of “translation repressor activity” indicates that the host directly inhibits the ribosomal function of protoscoleces and blocks its protein synthesis. The synergistic effect of these mechanisms indicates that *B. javanica* flavonoids form a multi-target antigenic effect by synergistically inhibiting the RNA stability, translation efficiency and immune escape ability of protoscoleces. KEGG pathway enrichment analysis further supported a multi-pathway synergistic mechanism. In immune defense, pathways such as “Influenza A” and “Toxoplasmosis” were activated, suggesting the host employs cross-defense strategies involving antiviral and anti-intracellular parasite immunity. In RNA metabolic intervention, the “RNA degradation” pathway was significantly enriched, which is functionally linked to the integrator complex, potentially accelerating protoscolex mRNA decay. In stress regulation, the “MAPK signaling pathway” was activated, potentially modulating host inflammatory and apoptotic responses against the parasite. Concurrently, enrichment in the “Protein processing in endoplasmic reticulum” pathway suggests disruption of parasite protein homeostasis (Figure 7).

The synergistic effect of these pathways reveals that *B. javanica* flavonoids form a multi-dimensional defense system to limit the infection of protoscoleces through immune response enhancement, RNA metabolism targeted intervention and stress signal network integration (Table 3).

**Table 3:** Proteomics-based target screening.

Features	baseMean	log2FoldChange	lfcSE	Stat	*p*-value	Gene symbol
A0A068X521	69866.28107	−1.84459	0.24860	−7.419	1.172151e-13	EgrG_002054100
A0A068WJN6	19325.62855	−2.78088	0.38706	−7.18463	6.73858e-13	EgrG_000646700
A0A068WQX2	350477.92356	1.67464	0.26306	6.36587	1.94177e-10	EgrG_000340400
A0A068WMY4	92222.18661	−2.585786	0.40643	−6.36212	1.98977e-10	EgrG_000432000
A0A068WMS7	289978.66074	−1.60366	0.25546	−6.27762	3.43803e-10	EGR_10089
W6U5H3	49209.61824	1.76903	0.29433	6.010377	1.85093e-09	EGR_08693
A0A068WPI4	84649.25659	1.91996	0.32234	5.95631	2.579983e-09	EGR_01203
W6UE48	226053.42302	−1.61111	0.29025	−5.550775	2.84406e-08	EGR_05999
W6UCL4	77958.29894	−1.55258	0.28568	−5.43464	5.49077e-08	EGR_06728
A0A068WC77	6998.79496	2.55140	0.48340	5.27808	1.30544e-07	EgrG_000759400
A0A068WNK8	393279.97992	−2.14044	0.41349	−5.17657	2.26000e-07	EGR_08219
W6U4N0	20141.91379	−1.50356	0.29254	−5.139671	2.75219e-07	EGR_09032
A0A068WHW5	204907.83591	−1.53618	0.30743	−4.99689	5.82618e-07	EGR_00974
D1MH11	521048.40448	−1.54174	0.32205	−4.78719	1.69130e-06	AgB3/2
A0A068X2H7	7511.37434	−1.54297	0.34259	−4.50383	6.6739e-06	EgrG_000733450
W6UD17	30212.12853	1.76644	0.40225	4.39143	1.12606e-05	EGR_08945
W6UQL2	77566.29085	−1.66190	0.37893	−4.38576	1.155846e-05	EGR_09427
A0A068WEJ3	7843.05677	−1.76278	0.41236	−4.27488	1.91240e-05	EGR_01500
A0A068WG98	98672.67860	−1.58597	0.37421	−4.23821	2.25312e-05	EGR_03009
A0A068WEU3	13446.09189	1.575810	0.38087	4.13741	3.51255e-05	EGR_02174
A0A068WAF1	6462.51710	1.62076	0.39450	4.10839	3.98425e-05	EgrG_000775450
W6U3X4	27067.73240	1.89883	0.46583	4.07626	4.57648e-05	EGR_09366
A0A068W9Z7	15980.69636	1.58333	0.39991	3.95920	7.52001e-05	EGR_00595
A0A068WJD1	3935.26746	−1.56225	0.53755	−2.90625	0.00366	EGR_06052
W6V1P3	173041.19157	−1.75447	0.73947	−2.37261	0.017663	EGR_05275

### 3.6 Molecular docking

Thirty-three pairs of flavonoid-protein interactions with binding energy < −9 kcal/mol were screened out by molecular docking. Among them, Hesperidin, Cyanidin 3-rutinoside cation, and Luteolin 4 O-glucoside (−10.3, −10.2, −10.0 kcal/mol respectively) had the strongest binding with the key protein A0A068X2H7. For the key protein W6USQ6, the strongest binders were dichamanetin, catechin 7-apioside, and cyanidin 3-rutinoside cation (−9.8, −9.8, −9.5 kcal/mol respectively). Thus, 16 flavonoids with the lowest binding energy were further screened, such as Hesperidin, Cyanidin 3-rutinoside cation and Luteolin 4-glucoside. Visualization with PyMOL revealed that these flavonoids formed stable binding poses within the active pockets of their target proteins, primarily through hydrogen bonds and hydrophobic interactions with key residues (e.g., Arg, Asp., Leu). Furthermore, multi-target binding was observed for several compounds. For instance, hesperidin showed high affinity to multiple proteins (A0A068X2H7, W6V1P3, A0A068WMY4), and glabrone bound strongly to multiple targets (A0A068X2H7, W6USQ6), suggesting their potential for multi-target action against the parasite (Tables 4, 5).

**Table 4:** The molecular docking results of the compound molecules and the differential proteins.

Protein name (UniProt ID)	Compound name	Binding energy kcal/mol
A0A068X2H7	Hesperidin,	−10.3
A0A068X2H7	Cyanidin 3-rutinoside cation,	−10.2
A0A068X2H7	Luteolin 4 O-glucoside	−10.0
A0A068X2H7	Dichamanetin	−9.8
A0A068X2H7	Scolymoside	−9.8
W6USQ6	Dichamanetin	−9.8
A0A068X2H7	Rhoifolin	−9.6
A0A068X2H7	Catechin 7-apioside	−9.8
A0A068X2H7	Myricetrin	−9.6
W6USQ6	Catechin 7-apioside	−9.6
A0A068X2H7	Neohesperidin	−9.6
A0A068X2H7	Glucoluteolin	−9.55
A0A068X2H7	Cyanidin 3-rutinoside cation	−9.5
A0A068X2H7	Erysubin F	−9.5
A0A068X2H7	Populnin	−9.5
W6USQ6	Cyanidin 3-rutinoside cation	−9.5
A0A068X2H7	Isosophoranone	−9.4
A0A068X2H7	Quercetin 3,4′-diglucoside	−9.4
A0A068X2H7	Cosmosiin	−9.4
W6V1P3	Dichamanetin	−9.3
A0A068X2H7	2-(3,4-Dihydroxyphenyl)-3,7-dihydroxy-4H-chromen-4-one	−9.3
A0A068X2H7	Catechin 7-apioside	−9.3
A0A068X2H7	Hirsutrin	−9.3
W6USQ6	Scolymoside	−9.3
W6USQ6	Glucoluteolin	−9.25
W6USQ6	Erysubin F	−9.2
W6V1P3	Hesperidin	−9.2
A0A068WMY4	Hesperidin	−9.2
A0A068X2H7	Glabrone	−9.1
W6USQ6	Glabrone	−9.1
W6USQ6	Neohesperidin	−9

**Table 5:** Flavonoid compounds with the lowest binding energy (Binding energy < −9 kcal/mol).

Compound name	Binding energy kcal/mol
Hesperidin	−10.3
Cyanidin 3-rutinoside cation	−10.2
Luteolin 4′-glucoside	−10.0
Dichamanetin	−9.8
Scolymoside	−9.8
Rhoifolin	−9.6
Myricetrin	−9.6
Neohesperidin	−9.6
Glucoluteolin	−9.55
Erysubin F	−9.5
Populnin	−9.5
Isosophoranone	−9.4
Quercetin 3,4′-diglucoside	−9.4
Cosmosiin	−9.4
Catechin 7-apioside	−9.3
Hirsutrin	−9.3

### 3.7 Observation on *in vivo* pharmacological effects and drug safety evaluation of myricetrin

Based on the results of the molecular docking of flavonoids and differential proteins, 16 monomer compounds were subjected to *in vitro* antigenic protoscolex experiments. Only C exhibited anti-activity against the primary protoscolex. (A) After the action of myricetrin on the primary cysticercus *in vitro* for 48 h, the cysticerci in the test group shrank, and the surface of the cysticerci showed collapse and local damage. In the drug concentration groups (2, 1.25 mg/mL), however, the cysticerci were transparent with visible internal organ structures, but had no abnormality in morphology or structure, indicating the minimum effective concentration of *B. javanica* flavonoids *in vitro* is 5 mg/mL. (B) The CCK-8 cytotoxicity test showed that after treating the BHK-21 cells with myricetrin for 48 h, the cell survival rate reached 95% in the test group with 5 mg/mL drug, and exceeded 98% in both test groups with 2 and 1.25 mg/mL drug. These results indicate the cytotoxic effect of myricetrin on cells is weak and does not inhibit the growth and survival of cells. (C, D) The mice were intraperitoneally injected with 100 mg/mL myricetrin for 7 consecutive days. The survival rate of the mice was 100%. Pathological sections of the organs and tissues (heart, liver, spleen, lung, kidney, and brain) were tested. The cells in each organ and tissue showed morphological and structural changes, but no substantial pathological damage, indicating the drug safety of myricetrin is relatively good. (E) Mice were infected with protoscoleces via intrahepatic injection to establish an infection model. 3-months after infection, the mice were intraperitoneally injected with myricetrin (5 mg/mL) for 21 consecutive days. Compared with the model mice, the cyst volume shrank, the cyst cavity collapsed, the proliferative layer structure of the cyst wall was damaged, dissolved and disappeared after the action of myricetrin. As the cyst volume decreased, the damaged liver cells gradually repaired, filling the damaged liver tissue, and the cyst adhered to the edge of the liver tissue. The test results suggest the *in vivo* effect of 5 mg/mL myricetrin can effectively kill the primary cysts and promote the repair of local liver tissues.

## 4 Discussion

*B. javanica* dried ripe fruit is a well-known medicinal herb (28) with anti-inflammatory, anti-malarial, and anti-tumor activities (29). Flavonoids are a group of natural polyphenolic compounds with various bioactivities. As one of the most effective components in traditional Chinese medicine (TCM), flavonoids have multiple pharmacological activities. The flavonoids in *B. javanica* are mainly composed of luteolin and its derivatives (30).

Echinococcosis seriously threatens human and animal health and causes significant economic losses. Surgery is the major treatment, accompanied by chemotherapy (31). Benzimidazoles are the main chemotherapeutic drugs, but they have limitations including low bioavailability, long-term toxicity, and unstable efficacy (32, 33). TCM has become a research hotspot owing to its low cost, few side effects and immunomodulatory effects. Its natural extract has a low risk of drug resistance, and the synergistic effect of multiple active ingredients is more advantageous (34, 35). TCM has become a research hotspot owing to its low cost, mild side effects and immue effects (36). Its natural extract has a low risk of drug resistance, and the synergistic effect of multiple active ingredients is more advantageous (37). TCM has a good application prospect in treating echinococcosis *in vivo* through various mechanisms. Almalki et al (38) found 30 and 50 mg/mL ginger extract acting on the protoscoleces of *E. granulosus* for 20 or 10 min achieved a 100% killing effect. Similarly, Yuan et al. (39) demonstrated that osthole (120 μmol/L) killed 100% of protoscoleces *in vitro* after 3 days. Furthermore, in a mouse model, a 6-week treatment with osthole (100 mg/kg) significantly reduced cyst wet weight compared to albendazole, while leveraging the safety advantage inherent to many natural compounds. Therefore, the development of natural anti-echinococcosis drugs with multi-target synergistic effect and safety is of significance to overcome the clinical bottleneck of high drug resistance and significant toxic and side effects of the existing chemotherapy.

The extraction rate was 10.72% by 80% ethanol, a solid–liquid ratio of 1:20, 60 °C for 30 min, and the ethanol concentration and solid–liquid ratio significantly affected the efficiency (*p* < 0.05). *In vitro* experiments showed that the crude extract had an inhibition rate of 85.88% on E.granulosus protoscoleces and induced apoptosis by activating Caspase-3. As reported in the literature that albendazole (ABZ) only achieved a 100% mortality rate of protoscoleces (PSCs) after 4 days of *in vitro* exposure at a concentration of 800 μg/mL, while ABZ at concentrations of 200 and 400 μg/mL required consecutive 9 days of exposure to reach a 100% PSCs mortality rate. In this study, the flavonoid extract of *B. javanica* flavonoids exhibited an 85.88% killing rate against protoscoleces at a concentration of 40 mg/mL after 24 h of treatment, with a significantly higher pharmaceutical efficacy than albendazole (40). SEM confirmed that it could destroy the surface structure. Toxicity assessment showed that the LD_50_ of CCK-8 was 1.43 g/kg in mice. Acute exposure did not cause organ damage, but the long-term effect of high concentration (10 mg/mL) may produce cytotoxicity. *In vivo* experiments confirmed that a 20 mg/kg dose could remove cysts and promote fibrosis repair, and the therapeutic index was better than that of synthetic drugs. Multi-omics integration provides a systematic basis for understanding the multi-pathway effects of natural products (41). A comprehensive analysis of the protoscolece protein samples revealed significant differences in protein expression patterns between the model group and the control group, providing important clues for understanding the effects of experimental treatments on parasite protein expression. Based on the molecular docking results for flavonoids and differential proteins, *in vitro* antigenicity experiments were conducted on 16 monomer compounds using E.granulosus protoscoleces. Only myricetrin exhibited inhibitory effects on E.granulosus protoscoleces. Plant-derived compounds show high translational potential as eco-friendly antiparasitic agents (42).

## 5 Conclusion

This study optimized the extraction of *B. javanica* flavonoids and confirmed their significant *in vitro* and *in vivo* anti-protoscolex activity against *E. granulosus* via apoptosis induction and multi-pathway regulation. Myricetrin was identified as the key active component with high efficacy and low toxicity. Limitations include *in vitro* bias and absent pharmacokinetic data; future studies will focus on myricetrin optimization and clinical translation.

## Data Availability

The datasets presented in this study can be found in online repositories. The names of the repository/repositories and accession number(s) can be found in the article/supplementary material.
